# Supplementary material for: Enhancement of the Surface Properties on Polypropylene Film Using Side-Chain Crystalline Block Copolymers
Source: Polymers (Basel). 2020 Nov 18;12(11):2736. doi: 10.3390/polym12112736 (PMC7698896; doi:10.3390/polym12112736)
Supplement: Supplementary file 1 [file polymers-12-02736-s001.pdf]

Supplementary Material

Enhancement of the Surface Properties on Polypropylene Film Using Side-Chain Crystalline Block Copolymers

Sho Hirai *, Patchiya Phanthong, Hikaru Okubo and Shigeru Yao

Research Institute for the Creation of Functional and Structural Materials, Fukuoka University, 8-19-1 Nanakuma, Jonan-ku, Fukuoka 814-0180, Japan

* Correspondence: shohirai@fukuoka-u.ac.jp; Tel.: +81-92-871-6631 (S.H.)

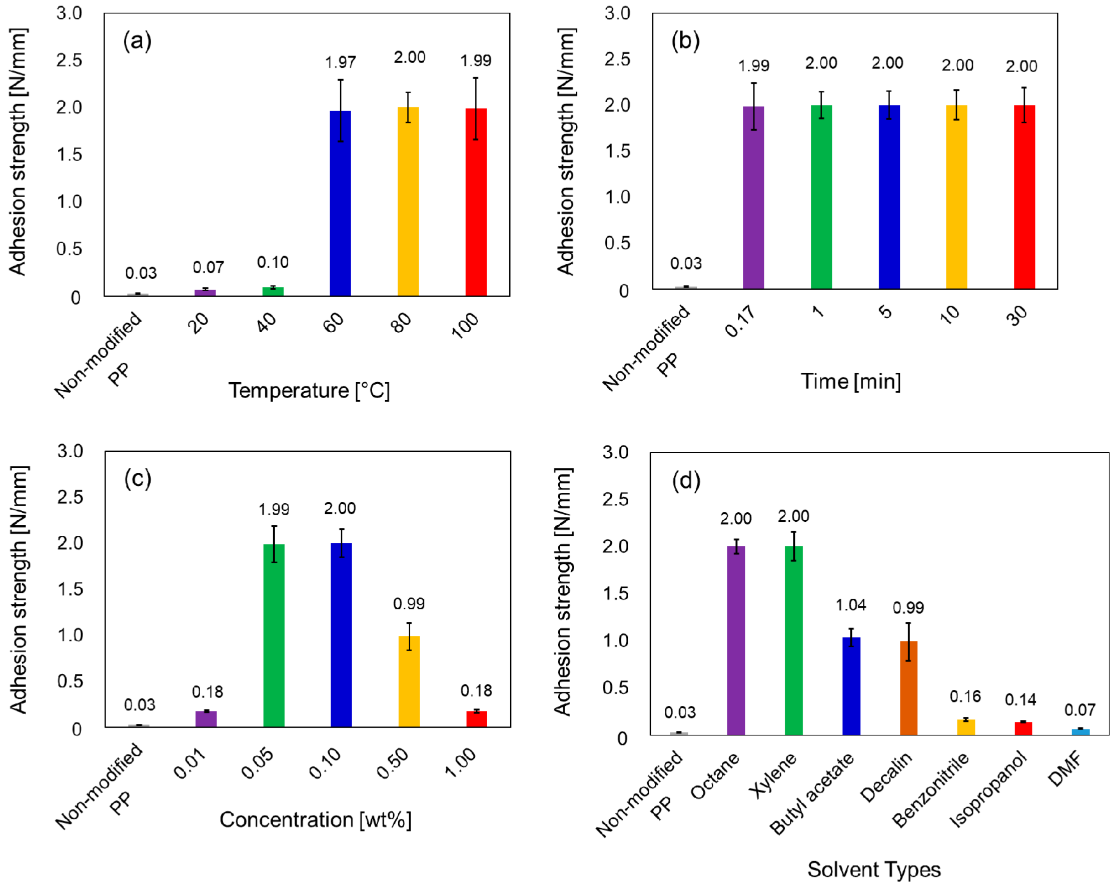

Figure S1. Adhesion strength of the non-modified PP and PP modified with the SCCBC evaluated by the T-peel tests: (a) 0.1 wt% of BHA-TBAEMA in xylene for 10 min at different dipping temperatures; (b) 0.1 wt% of BHA-TBAEMA in xylene at 80 °C with different dipping times; (c) BHA-TBAEMA at 80 °C for 5 min at different concentrations of BHA-TBAEMA in xylene solution; (d) 0.1 wt% of BHA-TBAEMA in different solvent types at 80 °C for 5 min.

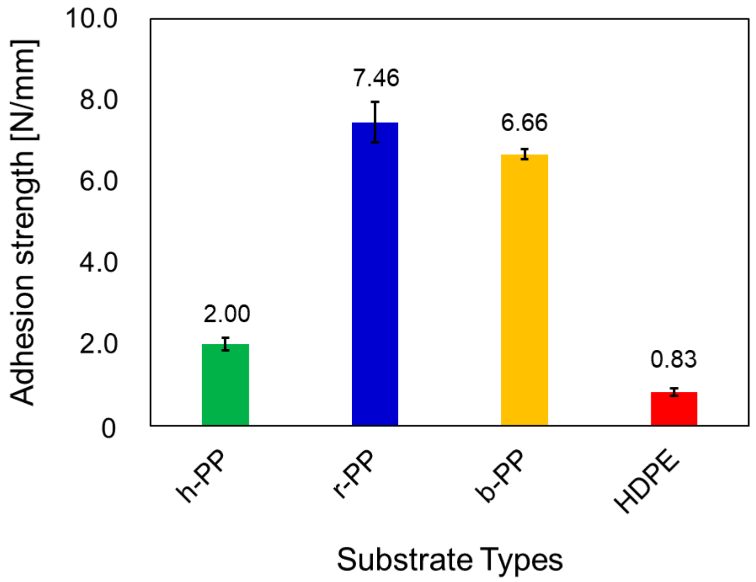

Figure S2. Adhesion strength of the modified h-PP attached with other substrates, which were also modified with BHA-TBAEMA under the optimized condition. These results were evaluated by the T-peel tests.
